# Is the preference of natural versus man-made scenes driven by bottom–up processing of the visual features of nature?

**DOI:** 10.3389/fpsyg.2015.00471

**Published:** 2015-04-23

**Authors:** Omid Kardan, Emre Demiralp, Michael C. Hout, MaryCarol R. Hunter, Hossein Karimi, Taylor Hanayik, Grigori Yourganov, John Jonides, Marc G. Berman

**Affiliations:** ^1^Department of Psychology, The University of ChicagoChicago, IL, USA; ^2^Adobe Systems, San JoseCA, USA; ^3^New Mexico State University, Las CrucesNM, USA; ^4^The University of Michigan, Ann ArborMI, USA; ^5^The University of South CarolinaColumbia, SC, USA

**Keywords:** esthetic preference, natural scenes, urban scenes, bottom–up processing, image features, perceived naturalness

## Abstract

Previous research has shown that viewing images of nature scenes can have a beneficial effect on memory, attention, and mood. In this study, we aimed to determine whether the preference of natural versus man-made scenes is driven by bottom–up processing of the low-level visual features of nature. We used participants’ ratings of perceived naturalness as well as esthetic preference for 307 images with varied natural and urban content. We then quantified 10 low-level image features for each image (a combination of spatial and color properties). These features were used to predict esthetic preference in the images, as well as to decompose perceived naturalness to its predictable (modeled by the low-level visual features) and non-modeled aspects. Interactions of these separate aspects of naturalness with the time it took to make a preference judgment showed that naturalness based on low-level features related more to preference when the judgment was faster (bottom–up). On the other hand, perceived naturalness that was *not* modeled by low-level features was related more to preference when the judgment was slower. A quadratic discriminant classification analysis showed how relevant each aspect of naturalness (modeled and non-modeled) was to predicting preference ratings, as well as the image features on their own. Finally, we compared the effect of color-related and structure-related modeled naturalness, and the remaining unmodeled naturalness in predicting esthetic preference. In summary, bottom–up (color and spatial) properties of natural images captured by our features and the non-modeled naturalness are important to esthetic judgments of natural and man-made scenes, with each predicting unique variance.

## Introduction

Previous research has shown that interacting with natural environments such as walking in a park or viewing images of nature can have a beneficial effect on memory, attention, and mood (Berman et al., 2008, 2012) as well as other psychological and physical health benefits (Ulrich, 1984; Kuo and Sullivan, 2001a,b; Cimprich and Ronis, 2003; Kaplan and Berman, 2010). Typically, the positive effects of interacting with nature are compared to the effects of interacting with urban or built environments. One reason that interacting with natural environments has been hypothesized to be beneficial comes from Attention Restoration Theory [ART; (Kaplan, 1995, 2001; Kaplan and Berman, 2010)]. ART claims that most natural environments do not tax top–down directed-attention mechanisms in the same way that many urban environments do, and they also provide softly fascinating stimulation that captures bottom–up involuntary attention mechanisms. It is this duality that is purported to make many natural environments restorative.

What is unclear, however, is why simply viewing pictures of nature vs. pictures of urban environments may lead to these benefits. Presumably, pictures of these environments would not capture attention in the same way as interacting with the actual environments, and yet researchers have found beneficial effects from simply viewing pictures of nature (Berto, 2005; Berman et al., 2008). The fact that pictures can elicit similar effects suggests that there may be low-level visual regularities of natural environments that may lead to psychological benefits. Alternatively, the very *semantic* idea of nature could be restorative. In other words, we might be “programmed” to benefit from low-level visual regularities in natural scenes in a bottom-up way, possibly imposed on our perceptual system through the process of natural selection. (See “savanna hypothesis” by Orians, 1986; Geisler, 2008).

In order to pave the way for future studies that will directly test this possibility, we first need to determine what the salient features that distinguish natural and urban scenes are and how these differential features may contribute to our preference for nature. Here we set out to determine the contribution of low-level statistical features versus semantics to esthetic preference judgments of natural vs. urban scene images. We do so by assessing the effects of bottom-up perceptions of naturalness that are driven by visual features of natural environments, e.g., the color green vs. perceptions of naturalness that cannot be predicted with certain low-level visual features and are possibly related to experience and semantic knowledge that affect one’s judgment more deliberately and slowly.

While preference may not necessarily correlate with the salubrious effects that are attributed to natural environmental interventions, it is an important starting point, since no single image is likely to produce restorative benefits on its own (i.e., it is likely that a set of images would be necessary). We already know that natural images are preferred over man-made scenes (Kaplan et al., 1972). In this study our main goal is to estimate how much of the preference of natural images is due to bottom–up visual regularities of natural scenes.

The first empirical research leading to models of statistical esthetics was probably conducted by Fechner (1876) concerning the golden ratio. He suggested that principles of esthetics could be inspected through statistical analysis, and that esthetic preference works in a bottom–up manner. Birkhoff (1933) formulated a simple “Esthetic Measure” based on his studies of polygons, and he proposed that the esthetic pleasure derived from an object is a direct function of the number of ordered elements (symmetry, equal sides, equal angles, etc.) and an inverse function of the number of complexity elements (number of sides, re-entrant angles, etc.) that attract a viewer’s attention. The simple square for example, had a very high degree of “Esthetic Measure” since it had both a high degree of order and a low degree of complexity. Eysenck (1957), on the other hand, argued that Esthetic Measure was the product and not the ratio of order and complexity because he found that preferred visual objects in his studies seemed to have both a high degree of order and complexity. More recently, information theory’s quantitative measures of complexity (e.g., Kolmogorov complexity) and measures of information redundancy (entropy) have been applied to esthetics (Rigau et al., 2008; Graham and Redies, 2010).

In addition, fractal-like statistics that are a statistical regularity in natural images have been shown to play a role in esthetic perception (Aks and Sprott, 1996; Spehar et al., 2003). For example, Aks and Sprott (1996) created chaotic patterns using mathematical equations with unpredictable solutions and showed that the fractal dimension (the extent to which the space is filled with details) and the Lyapunov exponent of the patterns (degree of unpredictibility of pattern production) correlate with esthetic preference of the pattern, and that preferred patterns have similar fractal dimensions to natural objects. Lastly, color-related features of scenes, including brightness, could be important to perception and esthetic preference (Mureika, 2005; Palmer and Schloss, 2009; Wallraven et al., 2009; Graham and Redies, 2010; Masuda and Nascimento, 2013). For example, Masuda and Nascimento (2013) showed that for daylights, colorful images of food counters are perceived as more natural under illuminants with an average correlated color temperature (CCT) of 6040 K and are most preferable under illuminants with an average CCT of 4410 K.

Another dimension of the scenes that is of interest in our study is the perception of naturalness. In parallel to esthetics, the quantification of statistical regularities in nature and how they are sensed or perceived have been the subject of interest for many researchers in computer vision (Ruderman, 1994; Huang and Mumford, 1999; Oliva and Torralba, 2001; Torralba and Oliva, 2003; Fei-Fei and Perona, 2005), mammalian vision (Field, 1987; Baddeley and Hancock, 1991; Olshausen and Field, 1996; van Hateren and van der Schaaf, 1998), and also in the context of ART (Berman et al., 2014). In this study, we sought to investigate the involvement of statistical regularities of nature in the esthetic judgments of different environments. We attempted to isolate more bottom–up aspects of perceived naturalness predicted by visual features from other factors that affect perception of naturalness in a scene. We hypothesized that if we regressed the naturalness ratings of the scenes from the low-level visual features of the scenes, the predicted values would be related to the more bottom–up aspects of judgments of naturalness (i.e., the low-level visual features impact on perceptions of naturalness).

There are many ways to decompose images into low-level visual features or descriptors. In this study, we were specifically interested in edge-related visual features [such as total edge density (ED), non-straight edge density and straight (SED) that capture contrast changes in surfaces, borders, and shades], entropy that is related to the shape of the histogram of pixel values in the image, and color related visual features such as average hue, saturation, brightness, and their average variations (standard deviations) that capture the main spectrum of colors in the scene and their variations. This focus stems from our previous research (Berman et al., 2014), which has shown that these visual features can reliably predict the perception of naturalness in images of urban, natural, and mixed urban/natural environments.

The goal of the present study was to explore the relationships between these quantified color and spatial image features and people’s esthetic preferences for the images, and then to estimate how much of the preference of nature images is due to bottom–up visual regularities of more natural images compared to the other aspects of natural scenes that are not modeled by these low-level visual features. With this knowledge, we may be able to isolate the effect of low-level features that occur in natural environments on judgments of preference from the more top–down semantics of naturalness. This information could then be used in the design of built environments to motivate interaction with nature and also improve psychological functioning.

## Materials and Methods

### Participants

All participants consented to voluntary participation via the guidelines established by the Institutional Review Board at the University of South Carolina and New Mexico State University. A total of 52 participants were enrolled in the experiment (26 female, mean age = 21.1). All participants reported normal or corrected-to-normal vision, and none reported any psychological or physical deficits that would exclude them from participation.

### Materials

Three hundred and seven images consisted of a spectrum of natural to man-made scenes (scenery of Nova Scotia, urban parks from Annapolis, Baltimore and Washington D.C., and pictures of Ann Arbor, Detroit, and Chicago) were used in the experiment. The pictures of Nova Scotia, Ann Arbor, Detroit, and Chicago were taken from Berman et al. (2008, 2014) and the images from Annapolis, Baltimore, and Washington, D.C., were provided by the TKF foundation and were utilized in Berman et al. (2014). All of the images can be downloaded from our PLoS ONE publication Berman et al. (2014): http://journals.plos.org/plosone/article?id=10.1371/journal.pone.0114572

This sample size provides sufficient statistical power for the study while keeping the behavioral experiments’ duration reasonable (less than 40 min). The images were in three different sizes: 512^∗^384, 685^∗^465, and 1024^∗^680 pixels. Importantly, all image features were normalized to the size of the images by being divided by the total number of pixels in the image.

### Procedure

Images were presented to participants using PsychoPy (Peirce, 2007) experimental software on a desktop computer. The experiment consisted of two tasks. In one task, participants were instructed to rate how much they liked each scene using a standard Likert scale from 1 to 7, with ‘7’ indicating a strong preference and ‘1’ indicating a strong dislike. During this procedure, images were shown for 1 sec and then removed from the screen and participants had up to 4 sec to rate each scene. This was to prevent long preference judgments, which could lead to contamination of decision with uncontrolled semantics or personal experiences.

In the second task, participants were instructed to rate how natural versus man-made each scene was. Again, a Likert scale was used with a range from 1 to 7, with ‘7’ indicating that the image was very natural and ‘1’ indicating that the image was very man-made. During this task, participants viewed the scene for 1.5 s before they were able to respond. This was to prevent hasty judgments about natural content before examining the image properly. After 1.5 s the scene stayed on the computer screen so that participants could view it until they made their response.

The order of presentation for 307 images was always randomized. For both tasks, participants responded using the numbers 1–7 on the computer keyboard. The order of performing the two tasks was counterbalanced across participants (26 participants did preference rating first, and the other 26 did naturalness rating first). Participants were instructed about each rating experiment separately and did not know about their second experiment before finishing the first rating. There was a 1- to 2-min rest between the two rating experiments.

### Behavioral Measures

Esthetic preference (Preference) for each image was calculated as the mean preference rating across all participants. Perceived naturalness (Naturalness) was also calculated as the mean naturalness rating across all participants for each image. The reason that ratings were averaged over all participants was that simple *t*-test comparisons showed that for none of the images the average preference from the 26 participants who did preference rating before naturalness rating was significantly different from the average preference rating from the 26 participants who did preference rating after naturalness rating (among all images, the closest to significance had *p* = 0.103, *t* = -1.692, df = 25). The same was true for naturalness ratings (among all images, closest to significance had *p* = 0.202, *t* = -1.312, df = 25).

### Quantitative Image Analysis Measures

#### Color Properties

**FIGURE 1:**
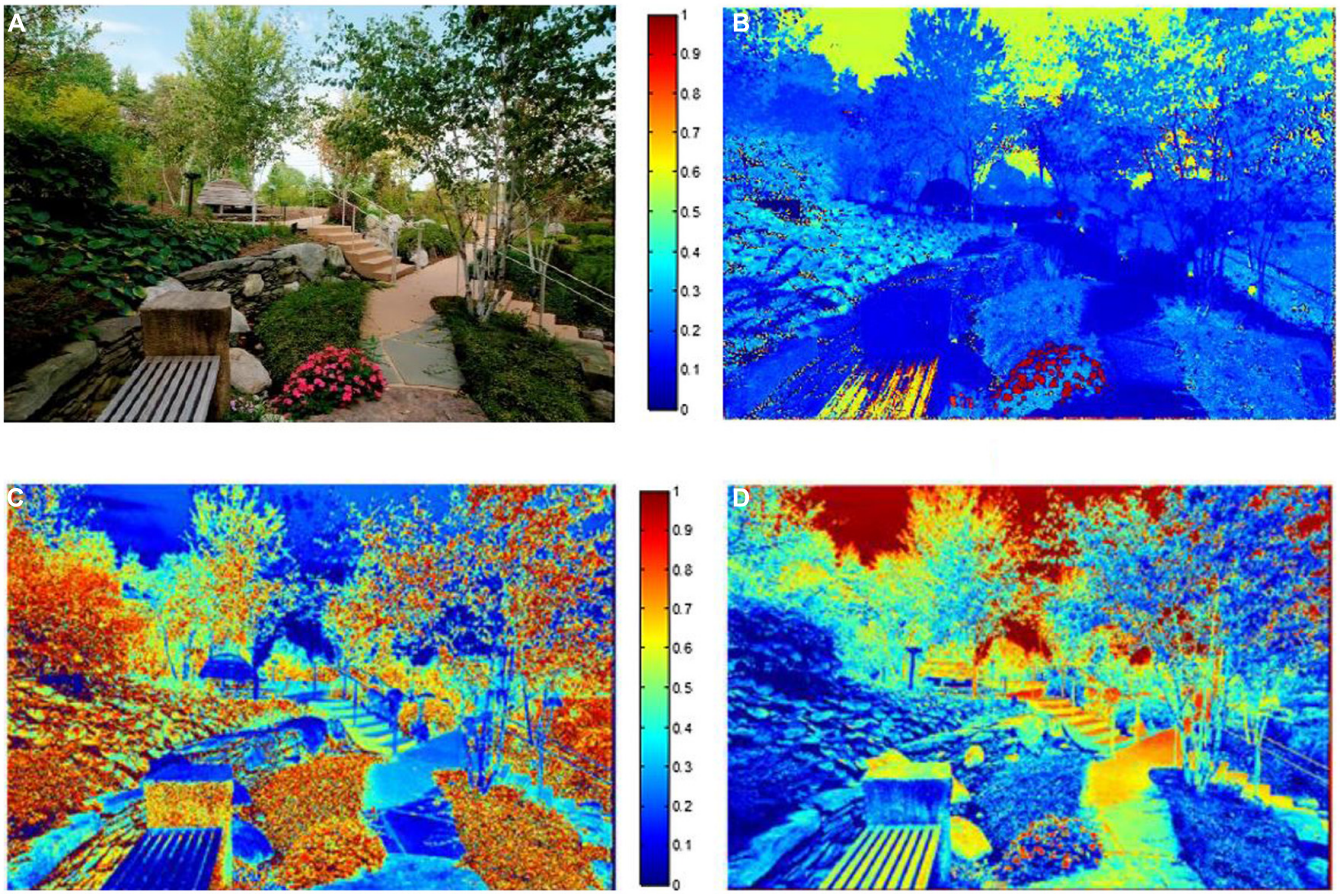
**(A) A sample image **(B)** Image’s hue map **(C)** Image’s saturation map **(D)** Image’s brightness map.** Pixels with hot colors have higher hue, saturation, and brightness in figures **B**, **C**, and **D**, respectively.

**FIGURE 2:**
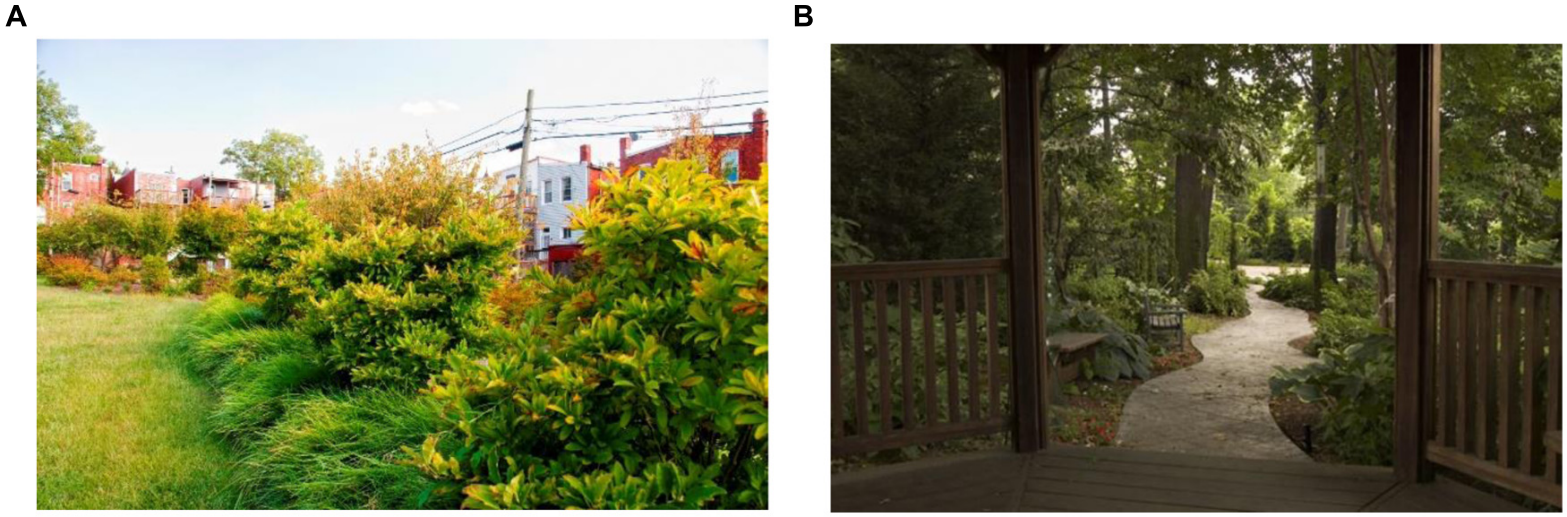
**Comparison of two images in their color diversity properties. (A)** Example of an image with high variation in saturation and medium variations in hue and brightness. SDHue = 0.19, SDSat = 0.38, SDBright = 0.28. **(B)** Example of an image with low variation in saturation and hue and medium variation in brightness. SDHue = 0.05, SDSat = 0.16, SDBright = 0.15.

In this section we describe the color features that were used in our analysis. Color properties of the images were calculated based on the standard HSV model (Hue, Saturation, and Value) using the MATLAB image processing toolbox built-in functions (MATLAB and Image Processing Toolbox Release 2012b, The MathWorks, Inc., Natick, MA, USA). (1) ***Hue*** is the degree to which a stimulus can be described as similar to or different from stimuli that are described as red, green, or blue. Hue describes a dimension of color that is readily experienced (i.e., the dominant wavelength in the color). We calculated the average hue across all image pixels and the average standard deviation of hue across all of an image’s pixels for each image. The average hue represents the hue level of the image and the (2) ***standard deviation of hue*** (SDhue) represents the degree of diversity in the image’s hue^1^. (3) ***Saturation***(Sat) is the degree of dominance of hue mixed in the color, or the ratio of the dominant wavelength to other wavelengths in the color. We calculated the average saturation of each image across all image pixels, as well as the (4) ***standard deviation of saturation*** for each image (SDsat). We also measured the overall darkness-to-lightness of a pixel’s color depending on the brightness of the pixel. This dimension of color is called (5) ***Brightness*** (Bright) or the value of the color. We computed the average brightness of all pixels for each image, as well as the (6) ***standard deviation of brightness*** in each image (SDbright). **Figure 1** shows hue, saturation, and brightness maps of a sample image in our experiment, and **Figure 2** compares two images in terms of their color diversity (SDHue, SDSat, and SDbright).

#### Spatial Properties

In this section we describe the spatial/structural features that were used in our analysis. A gray scale histogram of an image shows the distribution of intensity values of pixels that construct an image. Each pixel could have an intensity value of 0–255 (8-bit grayscale) and for a histogram with 256 bins, the probability value of the *n*th bin of the histogram (p_n_) shows the number of pixels in the image that have an intensity value of n-1 over the total number of pixels in the image. (7) ***Entropy*** of a gray scale image is a statistical measure of randomness that can be used to characterize part of the texture of an image using the intensity histogram. We used a simple definition of Entropy:

(1) $$Entropy\,\;=-\overset{256}{\underset{n=1}{\Sigma }}\left(p_{n}*\log_{2}\,\;p_{n}\right)$$

**FIGURE 3:**
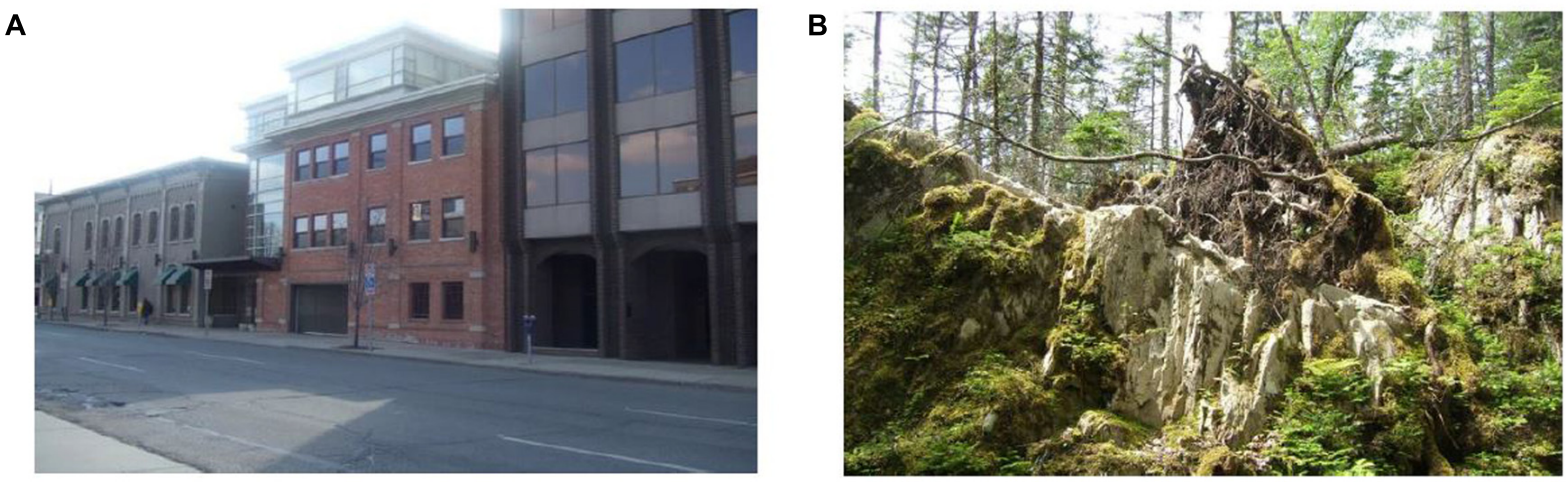
**Comparison of two images in their Entropy. (A)** Example on an image with low entropy, Entropy = 6.92 **(B)** Example on an image with high entropy, Entropy = 7.76.

Where p_n_ is the probability value of the *n*th bin of the histogram. Entropy shows the average “information” content of an image. The more the intensity histogram resembles a uniform distribution (all intensity values occur with the same probability in the image), the greater the entropy value becomes in the image. We calculated the entropy of the images as a measure of uncertainty or “information” content (versus redundancy) in the image’s intensity values. More comprehensive and sophisticated definitions or variants of image entropy have previously been applied for natural images (for example, see Kersten, 1987; Field, 1999; Chandler and Field, 2007) that are not in the scope of this study, as we focused on more simple features that are less computationally demanding and have straight-forward interpretations. In addition, our chosen features could be readily manipulated in visual stimuli and built environments by designers, architects, urban planners, etc. **Figure 3** shows a comparison of high versus low entropy in two images.

Another image feature concerned the spatial or structural properties of images provided by image gradients. An image gradient is a map of the image’s brightness intensity or color changes in a given direction. The points of discontinuity in brightness (rapid brightness or color changes) mainly consisted of object, surface, or scene boundaries, and fine details of texture in an image and are called *edges*. Images in this study (especially the more natural scenery) contain more complex detailed texture and fragmentations, which could lead to some complexities in edge detection.

The most commonly used method for edge detection is the Canny (1986) edge detection algorithm (see Klette and Zamperoni, 1996). This algorithm consists of five stages: first, blurring (or smoothing) an image with a Gaussian filter to reduce noise; second, finding the image gradients using derivatives of Gaussian operators; third, suppressing non-maximum gradient values; fourth, double thresholding weak and strong edges; and finally, edge tracking of weak or disconnected edges by hysteresis. This method is therefore less likely than the others to be influenced by noise, and more likely to detect true weak edges (see Canny, 1986). We used MATLAB’s built in function “edge” and set the method to “canny” to calculate the pair of thresholds to be used by the canny edge-detection algorithm for each image. MATLAB uses a heuristic method to calculate a “reasonable” pair of lower and upper thresholds for the Canny algorithm when the thresholds are not specified. Then, the same function was used for each image with thresholds specified as 20% below (high sensitivity threshold) or 60% higher (low sensitivity threshold) than those determined by MATLAB^2^. This was done so that we could weight faint and salient edges differently, with each pixel potentially having a value of 0, 0.5, or 1 depending on how sharp of an edge it belonged to. This was done in the following manner: pixels with a value of 0 were not identified as edges by the Canny edge detection algorithm at the high sensitivity threshold (i.e., pixels with a value of 1 were only detected as edges when using the high sensitivity threshold and not when using the less sensitive threshold (and therefore less salient edges); and pixels with a value of 2 were detected as edges with the lower sensitivity threshold and therefore were more salient. Finally, (**8)** ED was calculated for the image as the sum of total edge pixel values (i.e., 0 for non-edge pixels, 0.5 for faint edges, 1 for salient edges) over total number of pixels in the image.

Pixels belonging to straight edges (horizontal, vertical, and oblique lines) were also quantified so that SED and non-straight edge (curved or fragmented edges) density of images could be measured separately. Because of the complexity of the images, a typical Hough transform-based method could not detect straight lines accurately. Instead, we used a simple gradient-based connected component algorithm to detect straight lines in the images.

**FIGURE 4:**
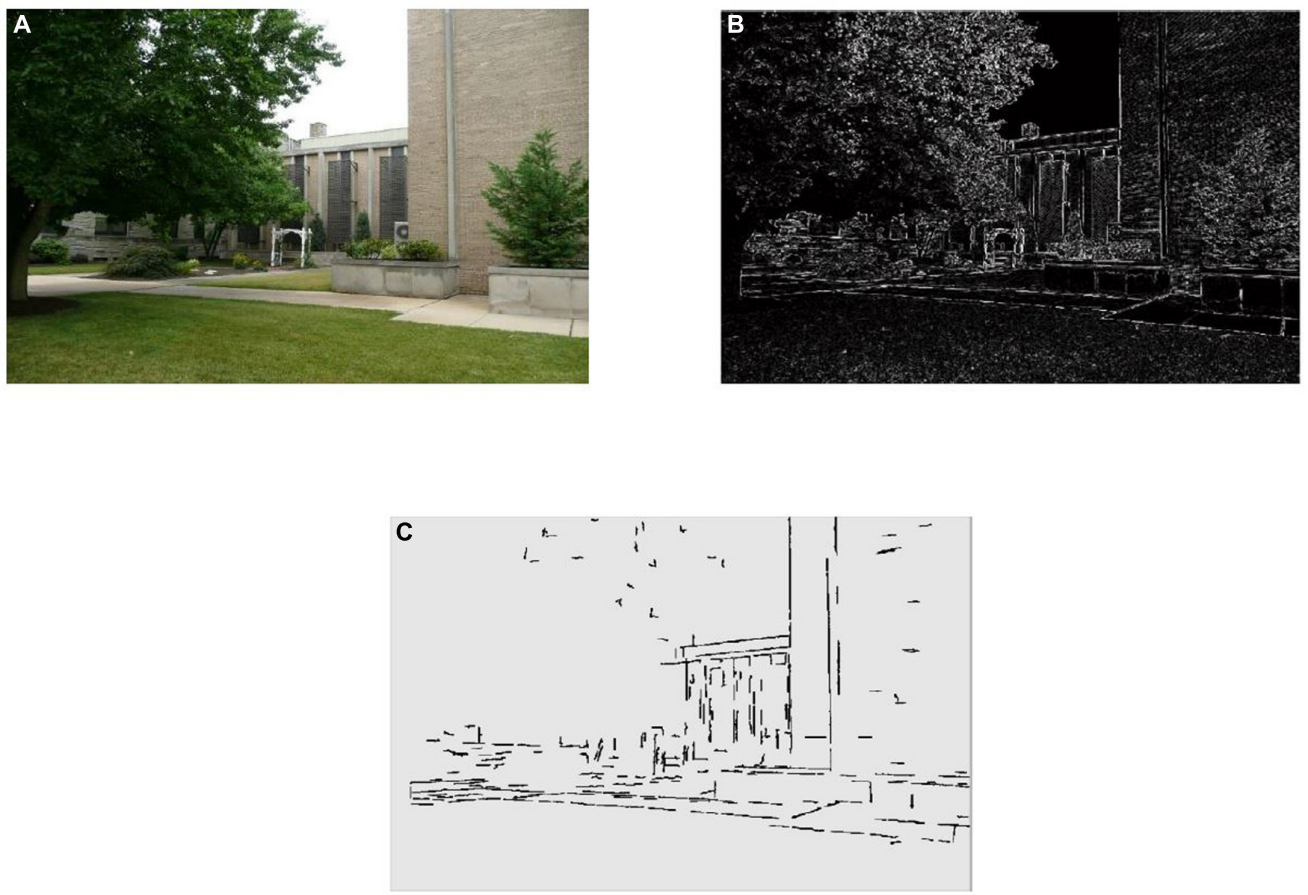
**(A)** Sample image, **(B)** the edge density (ED) map of the sample image created from salient and faint edges of the image detected using Canny edge detection, and **(C)** the detected straight edges of the sample image used to calculate SED.

First, the images were convolved with the derivative of a Gaussian filter in the X and the Y directions to compute the gradient directions for Canny edges. Second, each edge was assigned to one of eight directions based on its value of tan^-1^(G_y_/G_x_), where G_y_ and G_x_ are the *y* and *x* gradients. Third, the connected components for the edge pixels in each direction were determined and labeled using MATLAB’s ‘bwconncomp’ function. Finally, the Eigenvalues of the covariance matrix of the X and the Y coordinates of points for each connected component (edge) were used to compute the direction (the direction of the first principal component vector) and the straightness of the components. The first Principal component (PC) of the edge’ coordinates should be parallel to edge’s direction and the second PC captures the variability of edge’s coordinates perpendicular to its direction. Pixels of a connected component above a threshold of straightness (the singular value for the first principle component more than 10^4^ times larger than the singular value for second component) met the criterion of a “straight edge.” The number of pixels on detected straight edges over total number of image pixels was calculated as (9) SED for each image. **Figure 4** shows a sample image with its edge and straight edge maps.

**FIGURE 5:**
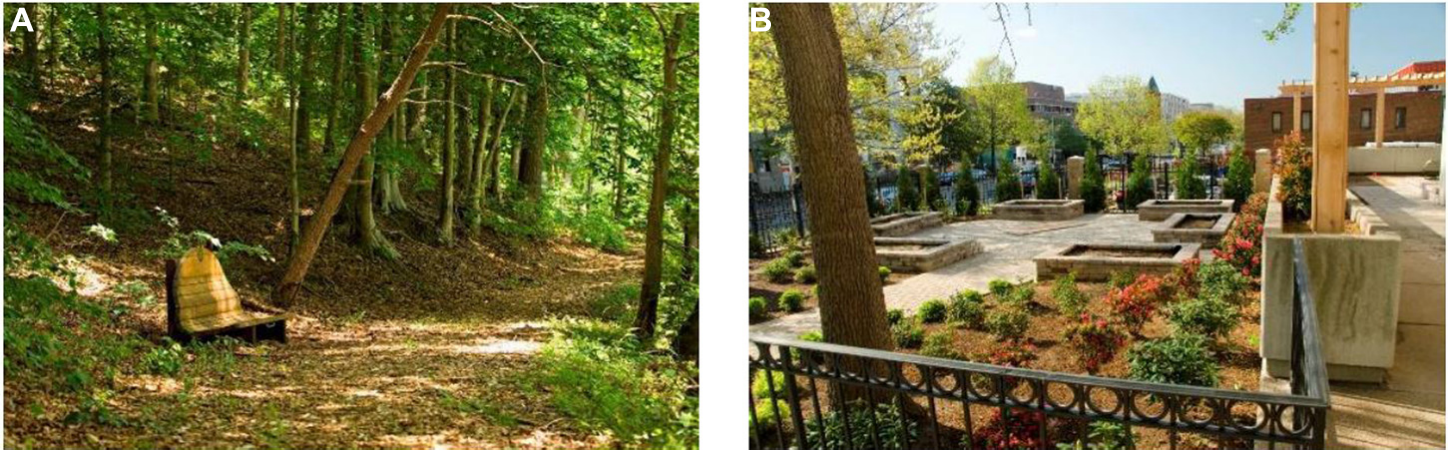
**Comparison of two images with different values of disorganized edge ratios: (A)** Example of an image containing high ratio of non-straight edges relative to total ED, DER = 0.84 **(B)** Example of an image containing low ratio of non-straight edges relative to total ED, DER = 0.41.

Lastly, in order to capture how many edges in an image consisted of curved or fragmented edges, the ratio of curved or fragmented edges (non-straight edges) density to total ED was calculated as a measure of (10) *Disorganized edge ratio* (DER) in each image. This measure captures part of the relatively more organized structure (greater in more man-made scenes) versus more chaotic or fragmented and non-straight edges (greater in more natural scenes) in the images, i.e., high DER indicates high disorganization. For example, **Figure 5** shows two images with high- and low-levels of DER, with their respective DER values included for comparison.

## Results

### Overview

In the results section, we first report the relationship of the quantified low-level image features with esthetic preference ratings using multiple regression. We then used our features to construct a model to predict naturalness based on the optimal linear combination of features. This was done by regressing naturalness ratings on the features and we did this to directly examine how modeled naturalness that is captured by bottom–up perceptions of naturalness relates to preference. Next, we confirmed the hypothesis that modeled naturalness is capturing bottom–up driven naturalness ratings. We did so by examining reaction times (RTs) when making preference judgments and found a positive relationship between RT and bottom–up naturalness (i.e., faster preference responses were more related to bottom–up naturalness), but the reverse relationship existed for non-modeled naturalness (i.e., slower preference responses were more related to non-modeled naturalness). Lastly, we assessed how naturalness modeled by structural visual features, naturalness modeled by color-related features, their interaction, and other aspects of naturalness that cannot be modeled with our features each uniquely contribute to esthetic judgment of scenes. In all of our analyses, we validated the generalizability of our models by utilizing a machine learning based classification analysis where we train a quadratic discriminant classifier to predict if an image was preferred by the participants.

### Low-Level Image Features Predicting Preference

The correlation matrix of preference and image features shows significant zero-order correlations between preference and Hue, SDhue, SDsat, Entropy, ED, SED, and DER (see Supplementary Appendix). However, some of these features were correlated with each other, which complicates the interpretation of the zero-order correlations as a feature’s correlation with preference may be confounded by another collinear feature. For example, SED is highly anti-correlated with DER as one would expect. Therefore, to assess the significance of each feature in predicting AP, we ran a regression of these features as variables in predicting preference. As can be seen in **Table 1**, low-level image features account for a significant proportion of variance in preference [adjusted *R*^2^ = 0.31, *F*(10,296) = 14.41, *P* < 0.05]. This result is comparable to others in this area (e.g., about 25% of variance of image preference judgments captured by statistics like sparseness for art images: Graham et al., 2010). Standardized regression coefficients and their confidence intervals show that lower hue and more saturation diversity in the image predict more preference while a greater number of straight edges predicts lower preference. The confidence interval of DER suggests a marginal positive effect for DER as a predictor of preference when other features are adjusted for. In fact, if we remove SED from the analysis, higher DER becomes strong predictor of preference, which is due to its high degree of shared variance with SED as mentioned before. In summary, some objective low-level features significantly predicted subjective preference judgments.

**Table 1:** Results of regressing esthetic preference on image features.

Predictor	Estimate	SE	*t* value	CI
Edge density	0.008	0.066	0.128	[-0.120 0.137]
Straight edge density^∗^	-0.382	0.064	-5.924	[-0.509 -0.255]
Hue^∗^	-0.177	0.061	-2.888	[-0.297 -0.056]
Saturation	-0.089	0.079	-1.125	[-0.244 0.066]
Brightness	0.085	0.051	1.66	[-0.015 0.185]
SDhue	-0.009	0.066	-0.139	[-0.140 0.121]
SDsat^∗^	0.214	0.069	3.096	[0.078 0.350]
SDbright	-0.031	0.054	-0.565	[-0.137 0.076]
Entropy	0.066	0.062	1.064	[-0.056 0.188]
Disorganized edge ratio	0.123	0.065	1.884	[-0.005 0.251]

Adjusted *R*^2^ = 0.31, *F*(10,296) = 14.41, ^∗^*P* < 0.05.

### Naturalness Ratings Predicting Preference

We also examined how perceptions of naturalness could predict preference. As one might expect, the naturalness of the image strongly predicted its preference, *R*^2^ = 0.52, *F*(1,305) = 340, *P* < 0.05, confidence interval = [0.66, 0.82]. This result indicates that images of natural scenes are more preferred than scenes with more man-made content. This is an expected result and replicates previous work by Kaplan et al. (1972).

### Low-Level Image Features and ‘Bottom–Up’ Perceptions of Naturalness

Next, in order to examine how preference is related to the variance of naturalness that is predicted by the low-level visual features versus the aspects of naturalness that is not captured by these features, we regressed naturalness on the visual features. The results of this regression are shown in **Table 2**. Replicating findings of Berman et al. (2014), these features explain a substantial amount of variance in naturalness ratings [*R*^2^ = 0.54, *F*(10,296) = 36.73].

**Table 2:** Results of regressing perceived naturalness on image features.

Predictor	Estimate	SE	*t* value	CI
(Intercept)^∗^	3.573	0.07071	50.53	[3.434 3.712]
Edge density^∗^	0.717	0.130	5.479	[0.459 0.974]
Straight edge density	-0.299	0.174	-1.713	[-0.643 0.044]
Hue^∗^	-0.249	0.091	-2.735	[-0.429 -0.069]
Saturation^∗^	-0.756	0.125	-6.028	[-1.004 -0.509]
Brightness	0.069	0.074	0.929	[-0.077 0.215]
SDhue^∗^	-0.220	0.094	-2.332	[-0.406 -0.034]
SDsat^∗^	0.244	0.099	2.462	[0.049 0.440]
SDbright	0.059	0.082	0.720	[-0.102 0.220]
Entropy	-0.021	0.085	-0.256	[-0.190 0.146]
Disorganized edge ratio^∗^	0.582	0.164	3.534	[0.257 0.906]

Adjusted *R*^*2*^ = 0.54, *F*(10,296) = 36.73, ^∗^*P* < 0.05.

We used the regression equation of naturalness = 3.573 –0.249^∗^*Hue* –0.220^∗^*SDhue* –0.756^∗^*Sat* +0.244^∗^*SDsat* +0.069^∗^*Brightness* –0.059^∗^*SDBright* –0.021^∗^*Entropy* +0.717^∗^*ED* –0.299^∗^*SED* +0.582^∗^*DER* +e as our linear model to calculate the predicted naturalness score (modeled naturalness) and its deviation from the naturalness rating (non-modeled naturalness) for each image. This way we could assess the degree to which preference of images is related to bottom–up naturalness that is captured by the visual features, assuming that naturalness that is predicted by this model is actually related to bottom–up perception of naturalness. This requires that the visual features capture most (almost exhaustively) of low-level visual information.

### The Effect of Bottom–Up Perception on Judgments of Naturalness

After separating naturalness ratings into modeled and non-modeled naturalness based on the low-level visual features, we hypothesized that if modeled naturalness is truly related to bottom–up processing (i.e., processing that is driven more by the stimulus features) of naturalness of an image, its effect on preference should emerge for faster preference ratings (i.e., faster RTs) since bottom–up processing tends to be a faster and more automatic process (Kinchla and Wolfe, 1979; Theeuwes et al., 2000; Delorme et al., 2004). This was tested by analyzing the interaction of modeled and non-modeled naturalness with the time to make a preference judgment (i.e., the RT for making a preference judgment) in predicting preference ratings for the images. RT for each image was the calculated average RT from each participant’s rating of preference from each image. RTs were *z*-scored within subjects to account for individual differences in the speed of preference judgments before being averaged over participants.

**Table 3** shows the results of the regression of preference on the interaction of preference RT with modeled naturalness (i.e., bottom–up perceptions of naturalness). As can be seen in the table, more naturalness captured by features strongly predicts more preference, i.e., images with more modeled naturalness are preferred over images of high modeled urbanness (the main effect). Importantly, the relationship of modeled naturalness with preference decreases as the RT of preference judgment increases in the images (the interaction). This suggests that when preference judgments are made quickly, those judgments are more reliant on the visual features of the images that drive naturalness ratings. Therefore, images that are more natural in a way that is predictable by this model are also rated more quickly by the participants. On the other hand, images that are more natural in a way that our model fails to predict (higher non-modeled naturalness) are the ones that are also rated slower and more deliberately by the participants. This is suggested by **Table 4** that shows the results of the regression of preference on the interaction of RTs with non-modeled naturalness.

**Table 3:** Results of the regression of esthetic preference on modeled perceived naturalness, RT, and their interaction.

Predictor	Estimate	SE	*t* value	CI
Modeled naturalness^∗^	0.453	0.051	8.896	[0.353 0.554]
Reaction time (RT)	-0.036	0.048	-0.752	[-0.131 0.058]
RT X modeled-naturalness^∗^	-0.247	0.048	-5.141	[-0.341 -0.152]

RT is Reaction Time, RT X modeled naturalness is the interaction term. Adjusted *R*^*2*^ = 0.34, *F*(3,303) = 54.23, ^∗^*P* < 0.05.

**Table 4:** Results of the regression of esthetic preference on non-modeled perceived naturalness, RT, and their interaction.

Predictor	Estimate	SE	*t* value	CI
Non-modeled naturalness^∗^	0.482	0.051	9.505	[0.382 0.582]
Reaction time	0.069	0.052	1.338	[-0.033 0.171]
RT X non-modeled naturalness^∗^	0.108	0.048	2.248	[0.014 0.202]

RT is reaction time, RT X non-modeled naturalness is the interaction term. Adjusted *R*^*2*^ = 0.25, *F* (3,303) = 35.01, ^∗^*P* < 0.05.

**FIGURE 6:**
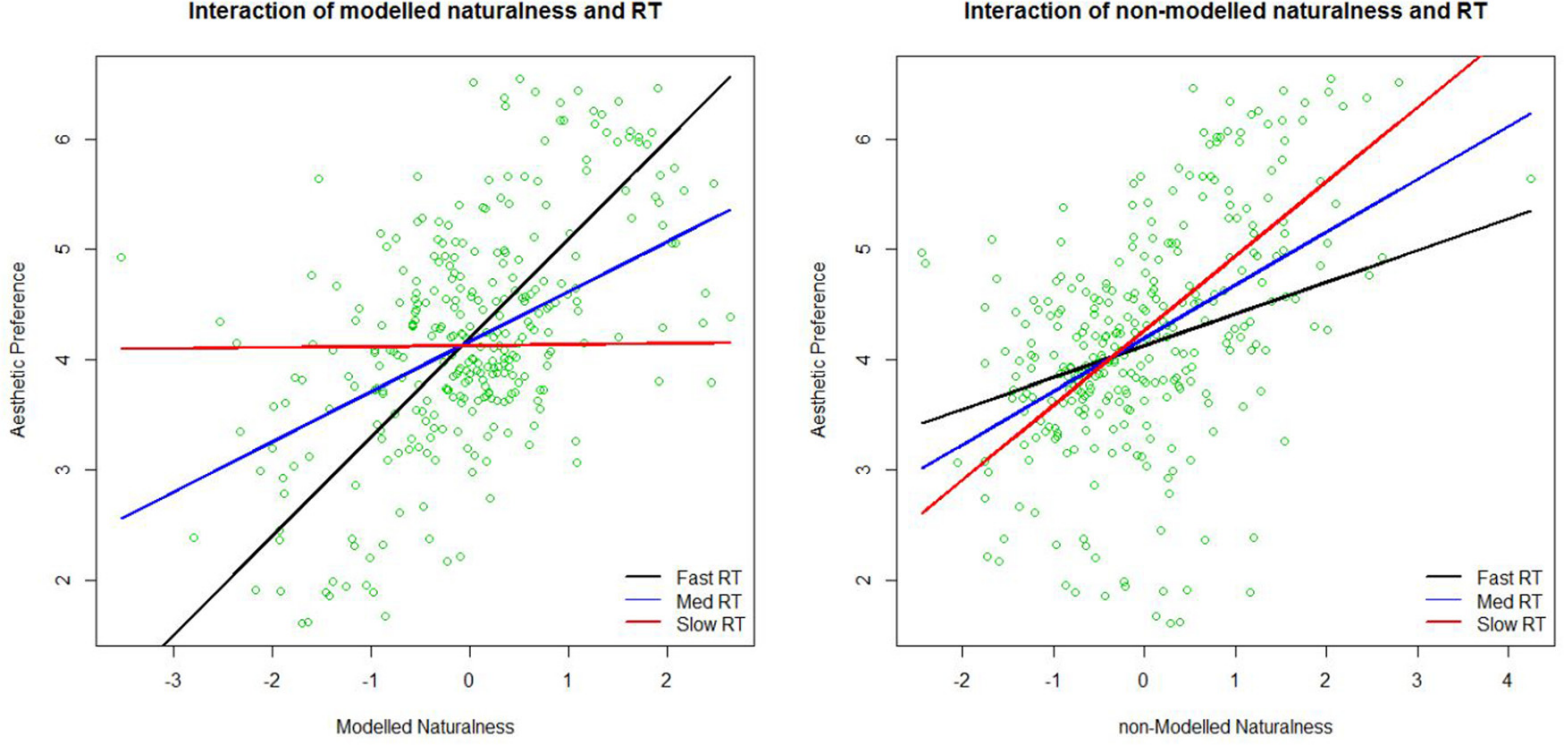
**Plots of the interaction of modeled naturalness and the reaction time (RT) to make the preference judgment **(left)** in predicting preference ratings, and the interaction of non-modeled naturalness and the RT to make the preference judgment **(right)** in predicting preference.** Lines show fitted regression lines of preference regressed on modeled naturalness **(left)** and non-modeled naturalness **(right)** at three different levels of RT. Med RT is at the mean RT and Fast and Slow RT are at mean RT ± 1.5^∗^SD of RT.

To illustrate this double dissociation better, we plotted the relationship between preference and modeled and non-modeled naturalness from **Tables 3** and **4** in **Figure 6**. Blue lines show regression lines relating preference to modeled naturalness (left) and non-modeled naturalness (right) when the RT of preference judgments are at the average RT. Red and black lines show regression lines relating preference to modeled naturalness (left) and non-modeled naturalness (right) when the RT of preference judgments are slow (mean + 1.5 SD), and fast (mean - 1.5 SD), respectively. As can be seen in the figures, modeled naturalness becomes a better predictor of preference in faster judgments (black line is steeper then red line in the left figure), whereas non-modeled naturalness becomes a better predictor of preference in slower judgments (red line is steeper then black line in the right figure). Therefore, we can claim that our features are modeling the more bottom–up aspects of naturalness important for esthetic judgment and the part of naturalness in the images that is not predictable by them is probably less related to bottom–up perception of nature. Whether the non-modeled aspects are associated with top–down perception of naturalness is beyond the scope of our data.

### Edge- and Color-Related Aspects of Naturalness in Predicting Preference

To isolate specific effects of color and structure, modeled naturalness was further decomposed to Naturalness-color (predicted naturalness based on only our six color features), and Naturalness-structure (predicted naturalness based on only our four spatial features), and also the interaction of these two components. **Table 5** shows the results of regression of preference on these two components and their interaction, as well as the non-modeled naturalness predictor.

**Table 5:** Results of regressing esthetic preference on color-related and structure-related bottom–up naturalness and non-modeled naturalness.

Predictor	Estimate	SE	*t* value	CI
Modeled-naturalness-color^∗^	0.362	0.081	4.454	[0.202 0.521]
Modeled-naturalness-structure^∗^	0.533	0.055	9.629	[0.424 0.642]
Non-modeled naturalness^∗^	0.480	0.043	11.164	[0.396 0.565]
Color X structure^∗^	0.207	0.092	2.237	[0.025 0.389]

Color X structure is the interaction between modeled-naturalness-color and modeled-naturalness-structure. Adjusted *R*^*2*^ = 0.47, *F* (4,302) = 69.36, ^∗^P < 0.05.

Our results suggest that people like both color-related regularities (less average hue and more diversity in saturation) and structure-related regularities (less straight edges and more disorganized edges), which tend to be more common in more natural scenes. Importantly, these features are more important in faster preference judgments. Additionally, the preference for these color-related and structure-related properties in an image increases preference not additively, but in an interactive way. Almost as important, people prefer the semantic idea of nature versus more man-made semantics, which may be captured by non-modeled naturalness (i.e., naturalness various that was not predicted by the low-level visual features).

### Quadratic Discriminant Classification of Esthetic Preference

To further assess the reliability/reproducibility of features and naturalness in predicting preference we performed a multivariate machine-learning classification analysis to predict preference. The classifier we chose was a quadratic discriminant (QD) algorithm, which has been implemented with great success to classify brain states and participants’ brain activity patterns (Yourganov et al., 2010, 2014; Berman et al., 2013). This classification analysis provides complementary information to the regression results as this classification analysis speaks directly to the reproducibility of the results and not just the effect-sizes. Also, because the classifier is non-linear and distinguishes classes with a non-linear surface, it provides additional information that is absent in a linear regression model.

We trained a multivariate machine-learning algorithm, the quadratic discriminant classifier, utilizing the low-level visual features to predict the preference of the images. Utilizing a leave-one-out cross-validation framework we could test how well the quadratic discriminant classifier could accurately predict the preference of the image.

Implementation of QD classification was performed using the classify function in the Statistics toolbox in Matlab (the classifier type was set to ‘quadratic’). The QD classifier uses a multivariate Gaussian distribution to model the classes and classify a vector by assigning it to the most probable class. The QD model contains no assumption of homoscedasticity, and instead estimates the covariance matrices separately for each class (that is, the variances of and the correlations between features are allowed to differ across high versus low-preferred images). This indicates that when implementing QD the two classes are separated by a non-linear curved surface.

We evaluated the success of the classifier using a cross-validation approach. A subset of images was used to train the classifier, and the image type based on a median split on AP (high preference versus low preference) was predicted for the images that were not included in the training set. At each iteration, two images (one high-preferred and one low-preferred) were held out for testing, and the remaining 305 were used to train the classifier; this process was repeated so that all combinations of high and low preferred images were determined by classification.

### Results of QD Classification of Preferred versus Not Preferred Images

For each combination of left-out high- and low-preferred images we computed whether the image type was predicted accurately. The proportion of images that were accurately predicted was our metric of *prediction accuracy*, our main measure of the efficacy of the classifier. We first introduced all 10 features for classification, and then excluded feature that dropped the classification accuracy. Our logic in the classification was to get maximum accuracy by omitting features that are redundant^3^. **Table 6** shows the prediction accuracy of the QD classifier using features only (Hue, Saturation, SDhue, SDsat, Entropy, SED, and DER (69.2%), using the modeled naturalness values only (61.9%), using the non-modeled naturalness only (66.9%), using modeled and non-modeled naturalness together (70.9%), and using features (Hue, Saturation, SDhue, SDsat, Entropy, SED, and DER) and non-modeled naturalness together (78.8%). In all cases chance is at 50% and also classification was above chance. This classification analysis demonstrates that features and semantics are each uniquely important and are reliable predictors of preference. The fact that features predict preference better than modeled naturalness shows some features are predictors of preference independent of their relationship to the perceived naturalness of the scenes.

**Table 6:** Results of quadratic discriminant classification of esthetic preference for images using different variables.

Variable(s) in classification	Prediction accuracy
Image features	69.2%
Modeled naturalness	61.9%
Non-modeled naturalness	66.9%
Modeled + non-modeled naturalness	70.9%
Image features + non-modeled naturalness	78.8%

## Discussion

We quantified the spatial structure and the color properties of a corpus of images with a spectrum of man-made and nature content by decomposing images that were rated on subjective preference and naturalness. Results from multiple regressions of preference on these low-level features showed that some of these features could significantly predict esthetic preference of the images and accounted for a large portion of the variance in preference. Specifically, lower SED (more non-straight surfaces, borders, and shades), lower hue level (lower hue means more yellow-green content rather than blue-purple content), as well as higher diversity in saturation (scene containing both low and highly saturated colors) predicted more preference, adjusting for the other visual features. In addition, and our linear model explained 31% of variance in preference ratings.

We also showed that individuals prefer scenes that are labeled as more natural. However, some of the image features predict preference above and beyond the semantic category of the images. In a relatively similar but more limited study, Kaplan et al. (1972) inspected how perceived complexity (rated by the subjects) and the content of images (natural versus urban) influenced esthetic preference of slides of nature and urban scenes. They found that nature scenes were greatly preferred to urban scenes and that complexity rated by participants (perhaps related to DER in this study) predicted preference within the natural and urban domains, but did not account for the preference for nature over urban slides.

Next, we used a linear regression and modeled naturalness by predicting it using visual regularities we found in the scenes rated as natural versus urban. These included higher ED and higher proportion of curved and fragmented edges, lower hue and saturation, less variation in hue levels, as well as more variations in saturation of colors. Using the estimates from our model of naturalness, we extracted the aspect of subjective naturalness that can be modeled by these visual characteristics. By introducing RTs of participants’ esthetic ratings on images into our analysis, this modeled naturalness was then shown to be a better predictor of esthetic judgments that are made faster and less predictive of preference in slow ratings.

After removing the low-level visual regularities of natural scenes, the remaining aspect of perceived naturalness is less likely to be correlated with bottom–up processing of preference as our analysis of RTs’ interaction with non-modeled naturalness showed that non-modeled naturalness becomes more predictive in more delayed preference judgments. This suggests that the other factors that affect naturalness judgments and are not modeled by our features, such as experience and semantic knowledge, may affect one’s judgment more deliberately and slowly. However, making firm conclusions about this is beyond the scope of our data.

One important question is why some of the visual features relate to perceived naturalness or esthetic preference and others do not? While we do not have empirical evidence to directly answer this question, we believe that some of the statistical regularities in natural and urban environments, which were uncovered here, make intuitive sense in terms of predicting naturalness. For example, shrubbery, trees, waterfalls, rocks and bodies of water have more yellow/green colors and also more defragmented or curvy surfaces compared to brick walls, streets, cars, fences, etc. Additionally, the variations in color saturation levels in a brown hillside are greater than that of a brown painted wall. These properties are in line with lower hue, higher ED, higher DER, less straight edges, and more SD in saturation; all of which were related to preference. In relation to previous work, fewer straight edges (or more non-straight edges) in a scene can lead to more complexity or more chaotic patterns that Kaplan et al. (1972) and Aks and Sprott (1996) previously associated with preference in scenes and visual patterns, respectively. In summary, we believe the features that were related to preference make sense from an intuitive perspective and also match with previous literature results. However, more empirical testing will be needed to understand the mechanisms behind preferences for these features.

The fact that predictable bottom–up perceptions of naturalness were related to preference suggests a few testable hypotheses in terms of which environments may be most restorative according to ART. ART claims that natural environments are restorative because they tend to place few demands on top–down directed attention, while simultaneously capturing bottom–up involuntary attention processes via soft fascination (i.e., modest attentional capture; Kaplan, 1995; Berman et al., 2008; Kaplan and Berman, 2010). By this rationale, it is possible that environments whose preference was determined more so by modeled naturalness (from the low-level visual features) may be more restorative than environments whose preference was determined more so by aspects of naturalness that could not be modeled with the low-level visual features. It could be theorized that images containing higher values of predictable (bottom–up) naturalness may be less taxing of top–down attentional mechanisms and thus more “softly fascinating.” For instance does interacting with man-made environments that resemble nature in these visual characteristics (for example fractal curves in architecture, using yellow–green colors with high saturation diversity in building facades, etc.) bring about some restorative effects of nature? Is naturalness that is more predictable also more effective in restoration than natural content that is not liked as fast and less predictable? These are all testable hypotheses that could help to inform ART and other theories of why interacting with nature is beneficial.

Fifty of the nature scenes that we used in this study have previously been shown to improve attentional resources and memory performance (Berman et al., 2008). The results we found here pave the way for our future work, which will focus on how the features we analyzed here might correlate with the attentional and memory benefits of viewing natural scenes, and how we might further inspect the driving low-level features that make interacting with nature restorative. Here too it will be important to separate the effects of the low-level features from that of the semantics (i.e., is it the low-level features that may improve cognitive performance or simply the very idea of nature that would improve performance).

It is important to emphasize again that restoration does not happen as a result of improvement in the mood by interacting with nature. In our previous work, we have found no relationship between improvements in mood and changes in memory and attention performance (Berman et al., 2008, 2012), indicating that participants do not need to enjoy the nature interaction to obtain the cognitive benefits. While mood and preference may not drive the cognitive benefits that are gleaned from interacting with nature, preference and mood may be driving motivational variables that would inspire one to interact with a natural environment. Here we have identified low-level image features that may increase this preference as well as the perceived naturalness of the environment. Our features are relatively easy to quantify and manipulate and have straight-forward interpretations for urban environment designers, architects, and planners. These features may motivate one to interact with an environment that could benefit them cognitively, and future work is aimed to determine whether these same features also act to improve cognitive performance directly, even when semantics are removed from the scenes.

## Conclusion

In this study, we aimed to explore whether the preference of natural versus man-made scenes is driven by the bottom-up processing of the low-level visual features of nature. We used 10 low-level visual features to predict esthetic preference in images containing a spectrum of urban to natural content. Our model successfully explained 31% of the variance in preference ratings. We also used these features to decompose the perceived naturalness of each image to its predictable (54% of variance modelled by the features) and non-modelled aspects and showed that bottom-up perceptions of naturalness (modelled by the image features) related more to preference when the preference judgment was faster (i.e., a shorter reaction time to make the preference rating). We also found that color-related and edge-related characteristics of nature interactively contribute to preferring one scene over another. Finally, to validate the generalizability of our models, we utilized a machine-learning classification analysis and were able to successfully train a classifier to distinguish liked (above median preference) and disliked (below median preference) images with almost 70% accuracy based solely on their visual features. Our results lend to the possibility that there may be low-level visual regularities of natural environments that we are “programmed” to gain benefits from (esthetic pleasure among others). Our findings have theoretical importance in determining why nature may be cognitively beneficial and may also have practical importance as these results could be used in the design of built environments to improve psychological functioning. Future studies may involve assessing the direct bottom-up effect of these features on the restorativeness of environments by manipulating these features in semantic-free visual stimuli.

## Conflict of Interest Statement

The authors declare that the research was conducted in the absence of any commercial or financial relationships that could be construed as a potential conflict of interest.

## Footnotes

1 There was the concern that since high hue (hue = [0.92, 1]) and low hue (hue = [0, 0.05]) surfaces essentially have the same color at high enough brightness and saturation (Red), treating hue as a linear scale could result in incorrect representatives of average hue and SDhue values. To check this, we changed the high hue red pixels with brightness and saturation above 0.5 to zero hue and recalculated the Hue and SDhue variables. However, these recalculated variables were almost identical to previous ones (*R*^2^ = 0.999 for both variables), hence no change was done to the analysis. The reason that SDhue and Hue are not dependent on the high hue red is that it happens with extremely low frequency in natural and urban photographic sceneries (On average, less than 0.01% of the pixels in our sample of images).

2 For example if MATLAB suggested a [0.1, 0.2] threshold pair for an image, we fed 0.8^∗^[0.1, 0.2] and 1.6^∗^[0.1, 0.2] to the Canny edge detection for finding faint edges and salient edges of that image, respectively (see Gonzalez et al., 2004). Importantly, the modifiers 0.8 and 1.6 were determined heuristically by OK and MB by screening the calculated edge maps of images for a range of different modifiers. This procedure is the same as was used in Berman et al. (2014).

3 The Classifier worked best with these features. Adding Brightness, SDbright and ED decreased classification accuracy. The fact that ED becomes redundant in classification analysis is probably because DER and SED and their quadratic and multiplicative terms capture all the information of ED.
