# Supplementary material for: Is the preference of natural versus man-made scenes driven by bottom–up processing of the visual features of nature?
Source: Front Psychol. 2015 Apr 23;6:471. doi: 10.3389/fpsyg.2015.00471 (PMC4407505; doi:10.3389/fpsyg.2015.00471)
Supplement: Supplementary file 1 [file Data_Sheet_1.DOCX]

**Appendix:**

Correlations matrix of image features and aesthetic preference (Figure S1) and their p-values (Figure S2) are shown here. Each colored square shows the correlation between variables that correspond to the row and column of the square (Figure S1) and the significance (p-value) of that correlation is indicated in Figure S2. Hot colors show values closer to 1 and cool colors show values closer to 0, meaning that dark blue show negative and yellow-red show positive correlations in Figure S1 and very dark blue show significance levels below .05 (p<0.05) in Figure S2 for these correlations. Pref is preference, ED is edge density, SED is straight edge density, and DER is disorganized edge ratio.

Figure S1. Correlations matrix of image features and aesthetic preference.

Figure S2. Significance levels (p values) of the correlations matrix of image features and aesthetic preference.
